# Shared flow and emotional synchrony through group instrumental improvisation: a feasibility study of music-based social connection

**DOI:** 10.3389/fpsyt.2025.1648873

**Published:** 2025-11-12

**Authors:** Edward A. Roth

**Affiliations:** Moores School of Music, Tilman J. Fertitta College of Medicine, University of Houston, Houston, TX, United States

**Keywords:** improvisation, emotional synchrony, shared flow, music therapy, mental health, group intervention, social bonding, music neuroscience

## Abstract

This feasibility and pilot study explored how group instrumental improvisation may foster emotional synchrony and shared flow, constructs linked with trust, bonding, and psychological well-being, and its therapeutic potential in mental health settings. Eight young adult participants, grouped by gender and musical background, engaged in two interactive conditions: instrumental music improvisation using digital mallet percussion instruments and a matched verbal improvisation task. Emotional synchrony and shared flow were assessed via validated scales and complemented by semi-structured group interviews to further capture participants’ experiences. All participants completed the full protocol, demonstrating the study’s procedural feasibility. Quantitative results indicated consistently high levels of shared flow and emotional synchrony across both conditions, with minimal differences between musicians and non-musicians. Qualitative findings highlighted participants’ sense of connection, attunement, and increased group cohesion, especially during musical interaction. Notably, musically inexperienced participants reported feeling connected and engaged despite no prior training, suggesting that structured improvisation may offer a low-barrier entry point for social connection in clinical settings. Feasibility of the study’s design is promising, as are the findings toward therapeutic applications in settings addressing social isolation, anxiety, or addiction recovery, where shared flow and bonding may serve as mechanisms of change.

## Introduction

Music seems to be a universal human behavior, an intrinsic aspect of human nature given its ubiquitous presence across all known human civilizations (1, 2). It has long served to strengthen emotional bonds, support rituals, and foster social cohesion (3). While theories of musical evolution vary, many scholars agree that music has served adaptive social functions, including the facilitation of group cohesion and emotional attunement (4). Extemporaneous musical interaction, improvisation, may thus offer a particularly resonant and evolutionarily grounded pathway to social bonding. Archaeological findings date music’s history to at least 35,000 years where bone flutes were discovered in what is currently southwestern Germany (5). Mithen (6) contends that music is actually much older and points to 300,000-year-old structures that were likely performance spaces and even, perhaps 350,000 years ago, hypothesizing that chanting and dancing may have emerged as a form of musical communication for bonding, infant care, and sexual display (7). One prominent theory of music evolution postulates that social groups with more sophisticated musical bonding practices likely enjoyed survival advantages over less musical groups (4, 7–9). In contemporary society, where the former U.S. Surgeon General declared loneliness and social isolation to be pressing public health issues with risks equivalent to smoking 15 cigarettes a day, related to mortality, (10), music remains embedded in shared social experiences of celebration, mourning, soothing, religion, and emotional connection (3). Much of early musical activity is believed to have been improvised (11, 12) before formal traditions were codified and passed down. Thus, extemporaneous musical interaction may offer a particularly natural and evolutionarily resonant means of fostering social connection, a premise explored in the current study. Today, these same mechanisms are increasingly relevant in mental health contexts where trauma, isolation, anxiety, and depression significantly disrupt interpersonal trust, co-regulation, feelings of safety, and ability to function in daily life. Music-based interventions, particularly those that emphasize social interaction and co-creation, may offer a unique and engaging path toward improved health and wellness in populations affected by disconnection, dysregulation, or adversity.

## Review of literature

### Music and social bonding

Dancing, singing, and other vocalizations were likely used toward motoric and emotional synchrony to bond large groups of individuals amongst our early human ancestors (3, 4, 9, 13–15). It is a much more recent phenomenon in human history to have individuals identified as expert musicians and even more recent to consider some individuals to be non-musicians and distinguish them from musicians (4, 7, 16, 17).

### Musicians vs. non-musicians

Comparisons between musicians and non-musicians appear frequently in the extant literature. Differences have been demonstrated based on expertise including biological differences. For instance, professional musicians have been shown to have significantly greater gray matter compared to non-musicians in brain regions associated with spatial, motor, and auditory functioning (18). Bi-hemispheric differences in grey matter were also observed between professional musicians and non-musicians in the cerebellum, supramarginal and angular gyri as well as the superior and inferior parietal lobe and middle temporal gyrus of the left hemisphere (19). To examine whether differences between non-musicians and musicians could be attributed to training, Hyde et al. (20) explored differences in brain structure between six-year-old children engaged in music training for fifteen months and those who received no training. At the onset of their study, differences in brain structure were not observed. By the end of fifteen months of musical training which consisted of half-hour private keyboard lessons, significant differences appeared in the corpus callosum, bilateral medial and superior frontal gyrus, right primary motor gyrus, and right primary auditory (Heschl’s) gyrus. Despite the many comparisons between musicians and non-musicians, to date, a universally agreed-upon definition of what constitutes the differences or demarcation points, remain elusive (21).

In one study, professional musicians were defined as those engaged in full-time music-making whose daily practice was greater than one hour, and participants who never played an instrument were defined as non-musicians (18). Grahn and Rowe (22) used musical training of greater than five years and ongoing musical experiences as inclusionary criteria for musicians, and Schulze et al. (23) also used training to define musicians by recruiting university students engaged in formal music instruction. More recently, Bumgarner (24) defined non-musicians as those having less than a year of music instruction and not having participated in music instruction or performance in the three years prior to their study. Similarly, Slater and Kraus (25) also defined non-musicians as those engaging in musical experiences less than three years in their lifetime and no participation in music-making in the three years prior to their study. What seems to be consistent across the human experience, regardless of musicianship level or stylistic complexity, is music’s capacity to foster communal bonding and emotional synchrony (4).

### Music, emotional synchrony, and flow

Such activity is elicited by music, in part, due to its temporal components (e.g., tempo, rhythm and meter) as they lead to the unconscious synchronization of movement (26, 27), and synchronizing movement to a “beat” promotes social bonding, cooperation, and trust (4, 28–32). These experiences are emotionally rewarding (31) and when engaged in pleasurable group music-making, individuals often report being in a state of “flow”, that is, a state marked by positive affect, high attention, perturbed sense of time, and loss of sense of self or “oneness” (33). The concept of flow was originally introduced by Csikszentmihalyi (34–36) and he described the experience as being in an optimal state, within an individual context. More recently, there has been an increasing interest in the study of optimal experiences resulting from shared flow and their relationship to emotional synchrony experienced during a group activity such as sports and music (37–42). A recent systematic review and meta-analyses of collective flow experiences within a music-performance context revealed that in a group setting, music facilitates flow through subsuming of individual goals for the collective good, application of individual skill to meet group challenges, and the coordination of individual contributions toward a coherent group performance (43). Given the known health benefits of social support (44), and the experiences of social bonding facilitated through shared flow from music experiences, Silverman and Baker (45) propose that flow could be considered a mechanism of change within a therapeutic context and provided models on which to build related research.

At the group level, most research around music, flow, and emotional synchrony thus far has been conducted using music listening or rote musical experiences. With few exceptions (46), studies of improvisation and flow, have been conducted using non-experimental designs (47). However, other constructs and applications have been studied experimentally in group improvisation such as social coordination through improvisational dance (48), joint attention behaviors through improvisational music therapy for children with autism (49), and a randomized controlled trial that found group improvisation to be an effective tool to treat depression in adolescents and adults with substance abuse disorders (50). The primary thrust of the current study was to examine the feasibility of the experimental design and examine music improvisation as it relates to emotional synchrony and shared flow. To do so, music improvisation was compared to verbal improvisation, which is described as being similar in structure, serving similar social purposes, and including similar everyday playful and creative behaviors (51, 52).

## Methods

### Study design

This study used a 2x2x2 mixed factorial design that compared musicians to non-musicians and interactive improvised music to interactive improvised verbalizing on the constructs of emotional synchrony and shared flow. Each participant served as their own control and the design was conceptualized to: 1) assess the feasibility of study procedures including experimental tasks and outcome measures; 2) compare the effects of music improvisation to verbal improvisation; 3) make comparisons between musicians and non-musicians; 4) compare participant experiences of shared flow and emotional synchrony; 5) examine participant experience in greater depth using a group interview process.

### Participants

Participants included young adults over the age of 18 grouped by gender and music background (N=8; two females and two males in each group; Group 1: Musicians; Group 2 Non-musicians). Following approval by the university’s human subjects institutional review board, participants were recruited and an informed consent process was completed, culminating in their consent to participate. Participants were selected into groups based on their musical background. Musicians were recruited from the instrumental jazz studies program at a large midwestern university in the United States and non-musicians were recruited from the broader student population at the same university. Students who were jazz studies majors were included due to their highly developed ability to improvise musically in comparison with non-musician controls. Although all participants were experienced improvisers, all of their primary instruments were something other than the instrument used to carry out the procedures of this study, and none had any experience with the instrument prior to this study. Inclusion criteria did not include a mental health diagnosis; this study was designed to assess feasibility with a non-clinical population.

### Procedures

Data collection took place on two separate days, one for each group. Physiologic measures were also obtained from these participants, and the results are reported, separately. Participants arrived in a private room in the library of the university’s music and dance building and were asked if they had any further questions about what would be expected of them and to verify that they signed the consent form. They were escorted to a large office across the hall from the library and instructions were provided regarding the general structure of the music improvisation.

The improvisations were carried out on three-octave (37 tones) MalletKat Pro digital mallet instruments (88), two of which were set to sound like marimbas which typically use wooden bars to produce tones, two of which were calibrated to sound like vibraphones which, unlike marimbas, use metal bars. The instruments appear similar to small xylophones and players strike foam pads that are organized as those on a vibraphone to trigger the appropriate instrument sounds. The intervention was facilitated by the principal investigator (PhD, MT-BC), a board-certified music therapist with over two decades of experience in clinical improvisation and music neuroscience research and over three decades of clinical work. Procedural fidelity was ensured through use of a consistent musical structure, standardized verbal instructions, and identical environmental conditions for both groups. Participants explored all of the sonorous capabilities of the instruments and were given approximately one-minute to do so. Following the exploratory period, participants engaged in a music improvisation using the form: A, where A indicates the whole group improvising simultaneously, and B-A, C-A, D-A, E-A, where letters B, C, D, and E indicate improvisations from each individual participant. Within this form, the group improvised simultaneously for four measures followed by each individual improvising for four measures. The individual improvisations comprised two two-measure phrases. Following each individual’s improvisation, the full group responded by imitating the last two measures of the individual’s improvisation by conveying some aspect such as the rhythm, tonal contour, emotional quality, or some other identifiable feature. Phrygian mode was used to provide tonal boundaries that sound pleasing, an important aspect of inducing flow (53), and has been reported to elicit sustained attention among improvisers (54). The macro-structure (in music, referred to as form) was used to provide participants with the experience of being heard and validated by attentively listening to each other and demonstrating such, by musically reflecting back what they heard from each participant (55, 56).

Following music improvisation, which lasted approximately 10 minutes, participants moved back to the private room in the library where they were electronically administered the flow and emotional synchrony scales on laptops to collect their responses (see below). After completing the questionnaires, verbal improvisation procedures were conducted.

The verbal improvisation procedures resembled a group discussion where participants individually “improvised” a short verbal phrase as would naturally take place in conversation while the rest of the group listened. When a participant’s verbal statements were concluding, another participant indicated that they wished to respond by gesturing in some way or just began speaking, again, mirroring what may naturally occur in a group conversation. To demonstrate empathy and that they were listening to the previous participant, they reflected something back from that participant’s comments such as the emotional tenor or actual verbal content. They then offered thoughts of their own and the improvisational verbal process continued, lasting approximately 10 minutes. After completion of the verbal improvisation tasks, participants were briefly interviewed to offer feedback about the experience.

### Measures

#### Feasibility outcomes

Because the design of the study is relatively novel, measures were included to examine the feasibility of the study design. Outcomes included our ability to recruit and enroll participants, the ability of participants to understand and perform study tasks, completion rates, duration of procedures to limit fatigue, adequacy of equipment and materials, and the appropriateness of chosen scales to measure the phenomena in question.

#### Emotional synchrony scale

The scale of Perceived Emotional Synchrony (PESC) (38) comprises 18 items that assess subjective, general social well-being, and shared emotions associated with participation in group experiences. The scale is anchored from 1 (not at all) to 7 (very much). Cronbach’s α for total score for this sample was.92, indicating a high level of reliability. Content of the PESC can be found in **Appendix A** in **Supplementary Material**.

#### Shared flow state scale

The Shared Flow scale (SFS) (41) is an adaptation of the Dispositional Flow Scale (57) measuring optimal, or peak, performance, originally with elite athletes. The SFS uses individual responses to shared group experiences and includes 27 questions across nine dimensions as originally described by (36): (1) Balance between challenge and skill; (2) Clear proximal goals; (3) Unambiguous and direct feedback; (4) Action-awareness merging; (5) Focused concentration on the current activity; (6) Sense of control over one’s actions; (7) Loss of self-consciousness; (8) Loss of time awareness or time acceleration; (9) Autotelic experience. The scale was anchored from 1 (not at all) to 7 (very much). Cronbach’s α for total score for this sample was.95, indicating a high level of reliability. Content from the SFS can be found in **Appendix B** in **Supplementary Material**.

#### Interview

Participants were interviewed as a complementary method to provide a more detailed account of their experiences related to improvising musically and verbally in relationship to the themes of emotional synchrony and shared flow. The interviews, conducted in groups, were also included to provide important feasibility information, such as identifying potential conflicts, weaknesses, or inconsistencies between the intended and actual participant experience (58). Data from structured interviews were obtained using a naturalistic inquiry framework, which is grounded in the belief that experience is context-dependent and constructed through shared meaning among participants (59–62) and were elicited by the following questions:

1. What stands out to you the most about this experience?
2. Do you recall moments when you experienced a sense of connection with an individual in the group?
3. Do you recall any moments when you experienced a sense of connection with the whole group?
4. If you felt a sense of bonding or connectedness, did you feel it more strongly in the music or verbal section, or about equally in both?
5. Were there moments when you felt particularly “out of sync” or disconnected from the group?
6. How were the experiences different between making music and talking?
7. Is there anything else we haven’t discussed that you’d like me to know?

The interviews allowed participants to raise additional questions and comments. Further inquiries were made of participants to facilitate the discussion by repeating questions, clarifying the intent of questions, and generally encouraging their responses. The interviews were video recorded and audio was extracted from the video files using Quicktime Player (91). Audio files were then uploaded to NVivo 12 Pro software package (92) to transcribe the interviews. Further transcription was required to correct semantic errors and this task was completed by the researcher.

Because the interviews were used as a complementary mechanism and not the main method of data collection, a brief inductive coding process was used to organize and understand practical information that could inform the implementation of a fully-scaled version of the study (62, 63, 90). Secondarily, a single level thematic analysis was used to reveal a cursory understanding of the participants’ subjective experiences. This analysis followed the general structure described by Braun and Clarke (64, 65), beginning with familiarization with the group interview transcripts, followed by identification of initial codes, recognition of recurring patterns, and the organization of these into provisional themes. Given the study’s focus on feasibility, the process was intentionally limited at this stage, emphasizing breadth over depth to capture salient experiences that might inform a fully powered version of the study, rather than pursuing a deeper interpretive analysis.

## Results

### Feasibility outcomes

Data are presented below for two primary outcomes, feasibility and subjective experience. As this study included feasibility considerations, recommendations for reporting the outcomes of feasibility studies by (66–68) informed data reporting and formatting of the analyses. The study also adhered to the updated Reporting Guidelines for Music-Based Interventions (RG-MBI) as described by Robb et al. (69), ensuring transparency of intervention design, delivery, and content. Given the small sample size, analyses of shared flow and emotional synchrony were conducted for feasibility purposes only. These analyses were not powered to detect statistically significant effects, rather, they were used to assess variability, acceptability of the measures, (e.g., would participants be able to respond to the prompts/questions based on their experiences with each condition), and to inform the assessment process of a fully-powered version of the study.

Of the eight participants who consented to participate in the study, all of them completed all study requirements including the interventions, questionnaires, and interview for a total completion rate of 100%. Completion of the full protocol lasted two hours 23 minutes for non-musicians and two hours two minutes for musicians. The onset of data collection began at approximately the same time of day for each group, 4p.m. for non-musicians and 5p.m. for musicians.

### Design, methods, and materials

All planned comparisons including non-musicians vs. musicians, females vs. males, and music vs. verbal conditions were achieved through the design of the study. The musical, technological, and inquiry materials all functioned as planned including the MalletKat instruments, amplifier, desktop computer and software, laptops and Qualtrics to administer the questionnaires, and the recording devices used to capture the interviews.

### Comparative analyses

Statistical analyses were completed using IBM SPSS v. 26 software (89). T-tests were used to examine between-groups and within-group differences using the Perceived Emotional Synchrony Scale (38) and Shared Flow Scale (41). Comparisons were made between musicians (n=4) and non-musicians (n=4); females (n=4) and males (n=4), as well as within-group comparisons for gender and music background following music and verbal improvisation conditions. On average, no statistically significant differences were observed between any of the variables on the PESC or SFS with alpha set at.05. As mean scores for all groupings of variables were higher than 4.00 (both scales used a range of 1 - 7), participants indicated that they experienced relatively high levels of emotional synchrony and flow. See **Tables 1** and **2**, respectively, for descriptive statistics. Interestingly, both groups reported consistently high ratings of shared flow and emotionally synchrony across both musical and verbal improvisation conditions, suggesting a generally strong receptivity to both forms of engagement.

**Table 1:** Perceived Emotional Synchrony Scale (PESC) means and standard deviations by gender and music background.

Group by Condition	Means	Standard Deviations
Females After Music	5.47	.84
Males After Music	5.56	1.10
Females After Verbal	5.36	.98
Males After Verbal	5.02	1.30
Non-Musicians After Music	5.96	.75
Musicians After Music	5.07	.92
Non-Musicians After Verbal	5.63	1.18
Musicians After Verbal	4.75	.90

**Table 2:** Shared Flow Scale (SFS) means and standard deviations by gender and music background.

Group by Condition	Means	Standard Deviations
Females After Music	5.96	.46
Males After Music	6.13	.54
Females After Verbal	5.93	.62
Males After Verbal	5.50	1.28
Non-Musicians After Music	6.27	.38
Musicians After Music	5.82	.49
Non-Musicians After Verbal	5.96	.84
Musicians After Verbal	5.46	1.13

### Emotional synchrony

### Shared flow

### Thematic analysis outcomes

All eight participants demonstrated the ability to reflect on, and articulate aspects of their experience related to connection, spontaneity, and co-creation. Interviews for both groups provided further evidence for the feasibility of the study design and procedures as participants indicated in their responses that they understood what they were asked to do in both musical and verbal conditions. Also related to feasibility, participants in both groups indicated that the verbal condition was “easier”, perhaps due to it being the second of two conditions. This is explored, along with other possible reasons, below in the participants’ comments.

There were two major themes in participant responses related to improvising musically and verbally including *Differences Between Music and Verbal Interactions* and *Feelings of Connectedness*. There was agreement in the thematic nature of responses across both groups, however, some differences existed between musician and non-musician participants and these are explored below using exemplary quotations.

#### Differences between music and verbal interactions

Musicians and non-musicians both noted that the experiences had similarities and differences, but some differences between their comments and questions were observed. Pseudonyms are used for confidentiality and were selected using an online service, *Name Voyager*, (70). For instance, musicians had more questions about the structure of the music than did non-musicians (who didn’t comment at all about the musical structure), such as “so, why did you decide to use Phrygian mode?” (Julia, musician). Another musician, Evan, pointed out that for him, “Probably the best for me, was the interaction with the marimbas. Because I mean, you know, I think it’s a different experience being a musician, but not being a percussionist” so, “there’s definitely a learning curve. I think it took us a little bit to like, you know, figure out how light the reaction time is” (Jordan).

Members in both groups noted that improvising musically likely had a priming effect that carried over to the verbal condition. Their observations included the comments “It was easier to have a discussion after the music performance because it’s kind of creating the same friendly atmosphere” (Jordan, musician); and “But I also think maybe it’s notable that, like, we did the music-thing first, right? Yeah. As we were, like, getting to know each other more. And then we opened up when we felt more comfortable during the talking part.” (Jennifer, non-musician). Other members who did not comment about this topic, in each group, did not contradict these observations and demonstrated agreement by nodding their heads or otherwise vocalizing.

Other observations were more directly related to the interactive aspect of the experiences. For non-musicians, Frank noted that he thought interacting musically avoided problematic interactions based on differences between group members when he stated, “but when you’re speaking with a group of people, that’s when a person with different opinions could get in the middle and cause problems, then you, I will say it would be easier, talking in music, you know? There will be prejudices while talking. And there wouldn’t be interruptions when somebody is talking musically.” Frank further stated “I think we’re all pretty good at communicating”, but for people who are “not like, necessarily, like social people, like they might feel more comfortable in the music situation”. Furthering this observation, Aaliyah, supported this idea by stating “Right, or at least to come out of your shell more because you’re like less, like, on the spot, maybe, when playing music”.

However, contrasting perspectives were shared amongst non-musicians, and pointed toward favoring verbal over musical improvisation in terms of its ease because they’ve been speaking for almost all of their lives or due to personality contributions, whereas, the study was the first time they had ever engaged musically with other people. For example, Aaliyah also stated “if my sister did this, (improvise musically) oh, she would be like sinking in her seat! I think it makes it a lot easier because we all seem pretty outgoing. I mean, basically, we’ve been talking since we were two!” That comment was echoed by Jordan, who stated “In the verbal, it’s more of like, oh, well, I should be this way or I should be that way. And that’s because we’re all speaking before we were playing music. So I think that’s kind of a little more ingrained. Whereas with the music, since I’ve developed a kind of a relationship with these people and myself, there was less of an expectation on, you know, I shouldn’t be this way or that way”.

Musicians were more uniform in their responses regarding their heightened comfort in music making over verbalizing. One example of this includes a comment from Lisa, “the music conversation was easier. It took us a while to get going, but once we figured out the groove, I think we all just super focused and especially … the last couple of minutes”. Followed by, “Yeah, I felt that, too” (Evan), and “yeah, yeah, and playing off each other” (Lisa).

#### Feelings of connectedness

Participants in both groups commented on feeling connected or bonded to other group members during both conditions. Musicians identified connecting with each other more during the music rather than verbal experience and also pointed out the effect of time-in-experience. “In the end … everyone started playing off of each other … I was listening to him for harmony and he was listening too. It wasn’t something I was doing prior to that” (Lisa). Julia agreed, stating “I think that (music improvisation) was, like, way more natural than the other connections I had with the group in general.” Interestingly, one of the musicians pointed out that during portions of the musical improvisation, they were all playing at the same time and that there wasn’t a good verbal analogue for that experience; “for me … (I felt more connected) during the musical, just because we were all consciously speaking in a way that’s like, you know, all constantly playing notes. Whereas here it was like, OK, I’ll respond to Evan. And then Lisa responds to Evan and it’s harder to do that when speaking.” This was supported by Evan, who also elaborated including speculation about the impact of personality; “I think for me with the verbal, I did feel it probably not as strong as the musical. For whatever reason, I’m not sure but the musical was more satisfying to get to that, to that, place. Yeah, I feel like it might also be a personality thing”.

Non-musicians seemed to indicate having strong feelings of connectedness during both experiences and considered several factors. Similar to what the musicians observed, non-musicians also pointed out the experience of making music simultaneously in back-and-forth discussion, observing, “really what we’re doing synchronized” (Simon) “so … everyone can see everyone’s… (taking turns) going around” (Jennifer) “yeah, and then making each other laugh!” (Aaliyah). “We were assigned to do this and then we were like, OK, let’s do this together and let’s try to … synchronize” (Frank). Aaliyah more directly addressed the issue by stating “I would say that … I felt more connected with everyone when we were talking. I mean, I felt connected when we did the music, but I also, like, really focused on what *I* was doing, like focusing on what *I* was going to have to repeat. But … when we were talking, I was more focused on what *everyone else* was saying.” Frank felt differently, saying “I will say the opposite, because … when you’re speaking…, unless you’re a good listener, you’re gonna be thinking of what you’re gonna say next.” In responding to a question about how it made her feel when Simon stated that he thought she was a good listener, especially during the verbal experience, Jennifer stated, “That makes me feel good. And it’s nice when you open up and you say something, you feel like someone else feels that way”.

## Discussion

The current study pursued two primary aims; 1) assess the feasibility of the study design including its tasks and outcome measures, and 2) better understand the participant experience as it related to emotional synchrony and shared flow. Overall, the feasibility of the study was confirmed, including the ability to recruit, enroll, and retain participants through the successful completion of the full study protocol.

The experimental tasks were feasible based on participants’ comments and ability to accurately complete each of the study’s conditions. In the music condition, the use of a simple 7-tone scale provided participants with a tonal structure by which to make music that was both interactive and pleasant sounding, and participants from both groups reported greater expressive freedom that increased as the experience unfolded. This suggests, perhaps, that music improvisation offered in this way provides immediate success, but breadth of creativity and purposive interpersonal expressivity may improve with experience. This phenomenon was similarly observed amongst groups of dancers with varying level of expertise, where dancers with greater expertise were able to improvise using greater creativity individually and more communicatively during joint improvisations (48). Participants were able to complete both questionnaires in the electronic format presented to them, accurately, completely, and in a timely fashion. Outcomes from the questionnaires seem to be in general alignment with information gleaned from the interviews, indicating that they are useful measures to assess the constructs of emotional synchrony and shared flow and the Cronbach values indicate satisfactory reliability for this sample frame. All mean scores exceeded 4.00, with all scores rounding to the nearest integers of five and six indicating that participants experienced relatively highly levels of emotional synchrony and shared flow during both conditions and across both male and female participants. This is particularly interesting as it suggests, if the findings were to remain consistent in a full scaled sufficiently powered version of the study, that prior experience with music improvisation may impact more nuanced aspects of the experience rather than fundamental responses that were shared by both musicians trained to improvise and non-musicians, alike. Although no statistically significant differences were detected, this was expected given the small sample size and exploratory nature of the analyses. The intent was not to determine efficacy, but to assess the feasibility of measuring shared flow and emotionally synchrony across conditions and to observe potential effect trends that could inform future study designs.

Thematic analyses of the interviews indicated that variations in participants’ experiences appeared to stem more from individual traits and preferences than from the nature of the intervention itself. This raises intriguing questions about the role of personality, openness, or improvisational comfort, perhaps even as it develops through experience, in shaping therapeutic engagement and outcomes; areas warranting further exploration in future designs.

The use of a simple mode, in this case Phrygian, provided enough tonal structure for musically naive participants to successfully produce music that was pleasing and sufficient tonal breadth to allow trained improvisational musicians to engage musically on their non-primary instruments. This finding is consistent with information in the extant literature on the introduction of improvisation in teaching and clinical scenarios (54, 56, 71–74).

Comparing improvising musically and verbally may be useful when selecting the types of experiences which could be employed toward personal health or use in therapeutic contexts (75). In this case, comparing improvisational music-making to verbal dialogue may provide information on which to make decisions about types of treatment, as experiences that lead to shared flow and emotional synchrony have been demonstrated to increase compassion for others as well as subjective well-being (76). These findings raise important questions about the relative effectiveness of music improvisation-based interventions compared to traditional verbal modalities. While this pilot was not designed to compare efficacy, the consistently high receptivity across conditions, and indications that individual preferences may shape engagement, suggest that music-based approaches merit continued investigation as a potentially equally effective, or even preferable, therapeutic pathway for certain populations.

### Limitations and future research

Because a convenience sample was used, all members of the group of musicians knew each other and had made music with each other as part of their university training, whereas none of the participants in the non-musician group had met each other prior to the study. Familiarity among participants in musical experiences may impact a sense of social closeness as Grettenberger (77) observed in a study comparing the effects of singing on feelings of connectedness between groups of participants who were, or were not, familiar with each other. Interestingly however, non-musicians in this study, despite not knowing each other beforehand, also reported strong feelings of connectedness during the music improvisation. This suggests, perhaps, that even brief, structured, facilitated improvisation may catalyze connection in newly formed groups.

The two conditions as implemented in this study were not isomorphic in structure as both conditions included periods of communication that featured individual contributions, but only the music condition included periods of time where all participants were playing in temporal synchrony as a group. If participants were to talk simultaneously, it would likely have been experienced as chaos, whereas the periods of synchronized music making in the current study were structured to elicit a sense of grounding (55) and social connection (56). Importantly, the fixed ordering of conditions, where musical improvisation always preceded verbal improvisation, may have introduced an exposure effect. This design limitation, driven by feasibility constraints in a small-sample pilot study, should be addressed in future research through counterbalancing the modality to better isolate the related effects. Expanding on this point, this observed effect may not merely reflect order; the musical improvisation may have actually shaped the emotional tone and interpersonal openness of the verbal improvisation that followed. Several participants described how the shared music-making helped them feel more comfortable and connected with their group partners, which may have carried into the verbal condition. This suggests that music improvisation may not only foster synchrony in the moment, but also prime subsequent interactions for deeper connection. Future studies might consider this potential cross-condition influence as a mechanism worth isolating in both design and analysis.

Although none of the current participants experienced difficulty completing either outcome measure, use of the PES short form (78) and short-form flow scale (SFS) (79, 80) should be considered in a large-scale multi-outcome version of the study to limit the amount of time required to complete study tasks. Relatedly, use of the Identity Fusion Scale (81), which measures the degree of “oneness” an individual feels with a group resulting in heightened personal agency and pro-group behavior, and the Vaux Subjective Social Support Scale (82), would both be useful measures in better understanding the links between social support experienced through music-making and improved physical and psychological well-being (44).

Although not employed at the feasibility stage, an adequately powered version of the study should counterbalance the order of interventions to control for exposure effects. The current participants were observant enough to note this in their remarks. While clinical improvisation is already a well-established and foundational method in music therapy, relative to the body of the extant literature, few studies have systematically examined its effects on shared flow and emotional synchrony using experimental designs. Beyond exploring the feasibility of the experimental design, the present study sought to examine improvisation, and its mechanisms of social and emotional engagement, within a controlled framework and in comparison to a standard form of interaction and therapy, i.e., verbal communication.

## Clinical implications and future directions

The findings from this pilot study add to a growing evidence base linking group improvisation, shared flow, and emotional synchrony to core processes in mental health. Clinical improvisation facilitated by a trained music therapist, can foster a relational co-regulation that is spontaneous, nonverbal and experiential, and emotionally rich, qualities shown to be especially important for populations affected by trauma, substance use, or social disconnection. Fachner and colleagues have contributed extensively to this area, demonstrating that clinical improvisation can modulate brain activity (83–86) and influence neurochemical processes related to emotion, empathy, and meaning-making (46, 87). More recent work using inter-brain analysis suggests that therapist-client dyads enter states of neural synchrony during live music-making, supporting the notion of shared consciousness as a therapeutic mechanism (93). These findings suggest that improvisation is not merely expressive but deeply integrative across biological, social, and psychological functioning. As such, it may be well suited for integration into group therapy programs, trauma-informed care, and community mental health models. Further research is warranted to bring to scale the current study and explore these possibilities across clinical populations and delivery contexts.

## Conclusion

The current study demonstrated an ability to yield useful information related to study design, recruitment, and enrollment. Current outcomes suggest that although differences in experience of flow and emotional synchrony exist between improvisationally trained musicians and non-musicians, those differences were statistically insignificant and subtle. Participants from both groups indicated that feelings of connectedness and flow increased over the duration of the music experience.

Considerations for further research include the use of short-form scales, counterbalancing the order of interventions, and familiarity among participants. Although in its early stages of research, the health benefits of social affiliation and shared flow may position the methods explored here as a promising pathway for supporting well-being among groups served by music therapists, particularly those experiencing dysregulation, impaired social connectedness, exposure to chronic stress, attachment disruption, or ongoing psychosocial stressors.

## Data Availability

The raw data supporting the conclusions of this article will be made available by the authors, without undue reservation.
