# Supplementary material for: Shared flow and emotional synchrony through group instrumental improvisation: a feasibility study of music-based social connection
Source: Front Psychiatry. 2025 Nov 12;16:1648873. doi: 10.3389/fpsyt.2025.1648873 (PMC12650769; doi:10.3389/fpsyt.2025.1648873)
Supplement: Supplementary file 1 [file Supplementaryfile1.docx]

Appendix A
Perceived Emotional Synchrony Scale

1. We felt stronger emotions than those we normally feel.
2. It seemed like we could read each other’s minds.
3. We felt that we were one.
4. We felt more sensitive to our emotions and feelings because we were surrounded by people who felt the same.
5. We felt a strong shared emotion.
6. We performed as one, like a single person.
7. We didn’t need words to express the feeling between us.
8. We felt a strong rapport between us.
9. We felt really united, almost melded into one.
10. What we were as a group was more important than what we were as individuals.
11. We felt more intense emotions because we all went through the same experience.
12. I felt as if I was transported out of myself, becoming part of the group.
13. It seemed to me as if we were a single person.
14. I felt a strong emotional bond between us.
15. We let ourselves get carried away by our emotions.
16. We communicated without words.
17. We shared a moment of unity.

We all felt a strong emotion.
