# Supplementary material for: Shared flow and emotional synchrony through group instrumental improvisation: a feasibility study of music-based social connection
Source: Front Psychiatry. 2025 Nov 12;16:1648873. doi: 10.3389/fpsyt.2025.1648873 (PMC12650769; doi:10.3389/fpsyt.2025.1648873)
Supplement: Supplementary file 2 [file Supplementaryfile2.docx]

Appendix B:

1. We knew that our skills would allow us to meet the challenge we were faced with.

2. We were doing things spontaneously and automatically.

3. Our goals were clearly defined.

4. It was really clear to us that we were doing well.

5. Our concentration was focused entirely on what we were doing.

6. We shared a feeling of total control.

7. We were not concerned with what others may have been thinking of us.

8. We felt that time was altered either speeded up or slowed down.

9. We really enjoyed what we were doing.

10. Our abilities matched the high challenge of the situation.

11. We felt that things were happening automatically.

12. We knew clearly what we wanted to do.

13. We were aware of how well we were performing.

14. We were completely focused on the task at hand.

15. We felt that we could control what we were doing.

16. We were not worried about what others may have been thinking of us.

17. We felt that the way time passed was different from normal.

18. We all found the shared experience extremely valuable and rewarding.

19. We felt we were competent enough to meet the high demands of the situation.

20. We performed automatically.

21. We knew what we wanted to achieve.

22. We were sure that in that moment we were doing really well.

23. We felt totally absorbed by what we were doing.

24. We felt in total control of our bodies.

25. We were not worried about the image we were presenting to others.

26. We felt like time stopped while we were performing.

27. The group experience left us with a good impression, a good taste.
